# A new age-related cutoff of medial temporal atrophy scale on MRI improving the diagnostic accuracy of neurodegeneration due to Alzheimer’s disease in a Chinese population

**DOI:** 10.1186/s12877-019-1072-8

**Published:** 2019-02-28

**Authors:** Mingqing Wei, Jing Shi, Jingnian Ni, Xuekai Zhang, Ting Li, Zilong Chen, Mengling Zhou, Liping Zhang, Zhongjian Tan, Yongyan Wang, Jinzhou Tian

**Affiliations:** 1 Neurology Centre, Beijing University of Chinese Medicine Affiliated Dongzhimen Hospital, Beijing, 100700 China; 2 Department of Radiology, Beijing University of Chinese Medicine Affiliated Dongzhimen Hospital, Beijing, 100700 China; 3 Institute of Basic Research in Clinical Medicine, China Academy of Chinese Medical Sciences, Beijing, 100700 China

**Keywords:** Alzheimer’s disease, Medial temporal atrophy scale, Posterior atrophy rating scale, Global cortical atrophy scale, Medial temporal-lobe atrophy index

## Abstract

**Background:**

Visual rating scales are still the most popular tools in assessing atrophy degrees of whole brain and lobes. However, the false negative rate of the previous cutoff score of visual rating scales was relatively high for detecting dementia of Alzheimer’s type (DAT). This study aimed to evaluate the diagnostic value of new cutoffs of visual rating scales on magnetic resonance imaging for discriminating DAT in a Chinese population.

**Methods:**

Out of 585 enrolled subjects, 296 participants were included and diagnosed as normal cognition (NC)(*n* = 87), 138 diagnosed as amnestic mild cognitive impairment (aMCI), and 71 as dementia of Alzheimer’s type (DAT). Receiver operating characteristic (ROC) curve analyses were used to calculate the diagnostic value of visual rating sales (including medial temporal atrophy (MTA), posterior atrophy rating scale (PA),global cortical atrophy scale (GCA) and medial temporal-lobe atrophy index (MTAi))for detecting NC from DAT .

**Results:**

Scores of MTA correlated to age and Mini-mental state examination score. When used to detect DAT from NC, the MTA showed highest diagnostic value than other scales, and when the cutoff score of 1.5 of MTA scale, it obtained an optimal sensitivity (84.5%) and specificity (79.1%) respectively, with a 15.5% of false negative rate. Cutoff scores and diagnostic values were calculated stratified by age. For the age ranges 50–64, 65–74, 75–84 years, the following cut-offs of MTA should be used, ≥1.0(sensitivity and specificity were 92.3 and 68.4%), ≥1.5(sensitivity and specificity were 90.4 and 85.2%), ≥ 2.0(sensitivity and specificity were 70.8 and 82.3%) respectively. All of the scales showed relatively lower diagnostic values for discriminating aMCI from NC.

**Conclusions:**

The new age-based MTA cutoff showed better diagnostic accuracy for detecting DAT than previous standard, the list of practical cut-offs proposed here might be useful in clinical practice.

## Background

As the number of the older patients grows, the increasing prevalence of dementia is becoming a major health problem among senior adults. Dementia affects 50 million people worldwide, with a new case of dementia occurring somewhere in the world every 3 s, the huge number of dementia patients brings a huge economic impact and burden of care [1]. Alzheimer’s disease (AD), as the most common cause of dementia, accounts for an estimated 60 to 80% of cases [2]. Present diagnosis of AD remains based on medical history, neuropsychological assessments, neuro-imaging, and laboratory tests. Although Pittsburgh Compound-B ([11C]PIB)-PET, as a molecular Imaging technique based on amyloid-beta pathology, could separate AD from the normal elderly with 100% specificity and 96% sensitivity, and was recommended as a diagnostic marker for AD [3].

However, there are some limitations of PIB-PET: firstly, the PIB-PET examination is expensive and many patients could not afford it; secondly, many hospitals don’t have the equipment to complete the PET examination in China; thirdly, there is a lack of a clear consensus on cut-off values for ‘positive’ or ‘negative’ amyloid of PIB-PET; finally, the PET examination is extensive and time consuming, which is hard to endure by patients with dementia, especially severe dementia. All the above disadvantages limit the routine application of PIB-PET in clinical practice. So far, magnetic resonance imaging (MRI) is still most commonly used for discriminating AD from other non-AD dementia in clinical practice, and it is able to estimate the progression rate of amnestic mild cognition impairment (aMCI) to AD [4]. In the 2011 AD diagnostic criteria, AD included typical AD (also named amnestic AD) and atypical AD (also named non-amnestic AD). The amnestic type is the most common syndrome presentation of AD dementia [5], which is characterized by atrophy of the medial temporal lobe (MTL), especially atrophy of the hippocampus and entorhinal cortex [6, 7]. In contrast, atypical AD may be presented with atrophy of the posterior cortical, posterior cingulate gyrus, precuneus and parietal lobes [8, 9].

Up to date, visual rating scales are still the most popular tools for assessing the degree of atrophy of whole brain and lobes. Medial temporal atrophy scale (MTA) is used for routine assessment of the medial temporal lobe [5]. Studies have reported that MTA showed optimal sensitivity and specificity for discriminating AD from non-AD cognitive impairment [10–12]. The posterior atrophy (PA) rating scale is used for the assessment of posterior atrophy [11], while the global cortical atrophy scale (GCA) was developed for assessment of global cortical atrophy. Previous studies have indicated that PA and GCA were useful scales for assessing regional brain atrophy and aiding AD diagnosis [11, 13, 14].

Medial temporal-lobe atrophy index (MTAi) is a new method for measuring the relative extent of atrophy in MTL in relation to the global cerebral atrophy, and is now considered to be more accurate than the MTA scale [15]. Scheltens, et al. have reported that patients at the age < 75 years with an MTA score ≥ 2, and at the age > 75 years with an MTA score ≥ 3 can be the optimal cutoff score for discriminating AD from controls7, and this cutoff criteria was widely used in clinical practice and research. However, the Scheltens’ cutoffs of MTA showed 18% false negative rate (FNR) in an Italian population study, and poor specificity (67%) for discriminating AD from the NC population [7, 16]. Moreover, the FNR of the Scheltens’ cutoff criteria was about 40% for detecting AD from NC in a Chinese population in our CHASE project [17]. Therefore, the current study sought to report our new age-based cutoff of visual rating scales and probe its diagnostic value.

## Methods

### Subjects

The CHASE project is a prospective cohort study based on a multi-center online case registration system from January 2013 to the present day. In this project 5357 subjects have been enrolled, all patients in this manuscript were included in the CHASE project. Chinese-speaking subjects aged 50 to 85 with memory complaints, who have completed the following diagnostic evaluation were enrolled between January 2013 and August 2017 in the memory clinic of Dongzhimen Hospital, Beijing University of Chinese Medicine, Beijing, China.

All participants underwent a routine clinical assessment, including detailed history taking, mental state examination, neurological examination, laboratory tests (i.e. thyroid function, folic acid levels, vitamin B12, and routine blood tests, among others) and neuroimaging. The neuropsychological assessment mainly included Mini-mental State Examination (MMSE), Instrumental Activities of Daily Living scale (IADL), Hachinski Ischemia scale (HIS), Hamilton Depression Scale (HAMD), Clock drawing test (CDT),the Adult Memory and Information Processing Battery story recall (DSR), Trail Making Test (TMT) and the Clinical Dementia Rating (CDR) score.

Subjects meeting criteria from the Mayo Clinic for healthy controls were allocated to the normal control (NC) group defined as [18], 1) Subjects without active neurological or psychiatric disease, (2) no psychotropic medication, (3) no medical disorder for which the disorder or its treatment could compromise cognitive function, (4) CDR = 0.

The following criteria were used to define aMCI: (1) memory complaints usually corroborated by an informant; (2) objective memory impairment (for age); (3) normal general cognitive function; (4) no or minimal impairment in activities of daily living; and (5) not sufficiently impaired in cognition and function, CDR = 0.5, and memory domain = 0.5 [19, 20].

The diagnostic criteria for dementia due to probable AD were based on the core clinical criteria of the National Institute on Aging —Alzheimer’s Association workgroups [5] and the CDR ≥ 1.

### MRI technique

All subjects received a standard dementia MRI scan at the department of radiology, Dongzhimen Hospital, Beijing University of Chinese medicine, on a 3.0 Tesla scanner (Siemens, Magnetom verio, Germany).

The scanning protocol included localizer scans and the sequences were showed as the following:

Spin Echo (SE) Sagittal (TR = 400 ms, TE = 8.9 ms, Slice = 23, Dist factor = 20%, Slice thickness = 5.0 mm, FOV = 243 × 320).

Fast low angle shot Axial (TR = 300 ms, TE = 2.5 ms, Slice = 23, Dist factor = 20%, Slice thickness = 5.0 mm, FOV = 219 × 320).

Fast spin echo (TSE) Axial (TR = 671 ms, TE = 110 ms, Slice = 40, Dist factor = 20%, Slice thickness = 3.0 mm, FOV = 207 × 320).

TSE flair (TR = 7800 ms, TE = 89.0 ms, Slice = 23, Dist factor = 20%, Slice thickness = 5.0 mm, FOV = 187 × 256).

TSE Coronal (TR = 500 ms, TE = 9 ms, Slice = 23, Dist factor = 20%, Slice thickness = 3.0 mm, FOV = 216 × 320).

### MRI readings

All of the MRI reading was conducted by two clinicians who were blind to the diagnosis and age of the subjects. A definite score was assigned when the two raters reached a consensus.

The MTA-score was rated on coronal TSE images at a consistent slice position according to the original study by Scheltens et al. [7], 0 = no atrophy; 1 = only widening of the choroid fissure; 2 = also widening of the temporal horn of the lateral ventricle; 3 = moderate loss of hippocampus volume (decrease in height); 4 = severe volume loss of hippocampus. The right and left hemisphere were rated separately, the MTA score being the average of these two values.

GCA scale was used to determine the mean score for cortical atrophy throughout the complete cerebrum and was scored on FLAIR images according to the Pasquier F [21], the score of the GCA scale ranged from 0 to 3, 0 = no cortical atrophy; 1 = mild atrophy: opening of sulci; 2 = moderate atrophy: volume loss of gyri;3 = severe (end-stage) atrophy: ‘knife blade’ atrophy.

PA rating scale was used to score the degree of parietal atrophy, and was rated in three different orientations Sagittal SE, axial FLAIR- and coronal TSE images. This scale was scored according to the Koedam ELGE., et al [13], 0 = no atrophy; 1 = mild widening of the sulci without evident volume loss of the gyri; 2 = substantial widening of the sulci and volume loss of the gyri; 3 = severe end-stage atrophy. When different scores were obtained, the higher one was used.

MTAi was measured according to Menéndez-González M, et al [15]. This method consisted of calculating a ratio with the area of 3 regions, tracing manually on one single coronal MRI slide at the level of the interpeduncular fossa: (1) the medial temporal lobe (MTL) region (A); (2) the parenchyma within the medial temporal region, that includes the hippocampus and the para-hippocampal gyrus—the fimbria taenia and plexus choroideus were excluded—(B); and (3) the body of the ipsilateral lateral ventricle (C). From this, we were able to work out the ratio of “Medial Temporal Atrophy index” at both sides as follows: MTAi = (A − B) × 10/C.

### Study approval

The protocol was approved by Dongzhimen Hospital, Beijing University of Chinese Medicine Institutional Ethics Committee. The study was undertaken in accordance with the principles of the Declaration of Helsinki. All the patients and responsible caregivers provided written informed consent.

None of the MRI rating scores were used in the diagnostic procedure.

### Statistics

SPSS 21.0 for Windows was used for the data analyses. Sex distributions in the three groups were compared using the chi-square test, mean age, education years, and neuropsychological test scores were compared by nonparametric tests (Kruskal–Wallis). Receiver operating characteristic (ROC) curve analyses was used to calculation of the optimal cutoffs of the visual rating scales for separating AD from NC, aMCI from NC, and AD from aMCI, the optimal sensitivity and optimal specificity was calculated using the highest youden index of different cutoffs. The optimal cutoffs of visual rating scales to discriminate AD from NC in different age groups was also calculated using ROC analyses. Multi-Linear-regression analysis was used to calculate the correlations between scores on each visual rating scale and age, education and neuropsychological variables. *P* values below 0.05 were considered statistically significant throughout the analysis.

## Results

### Demographic and neuropsychological variables

**Fig. 1:**
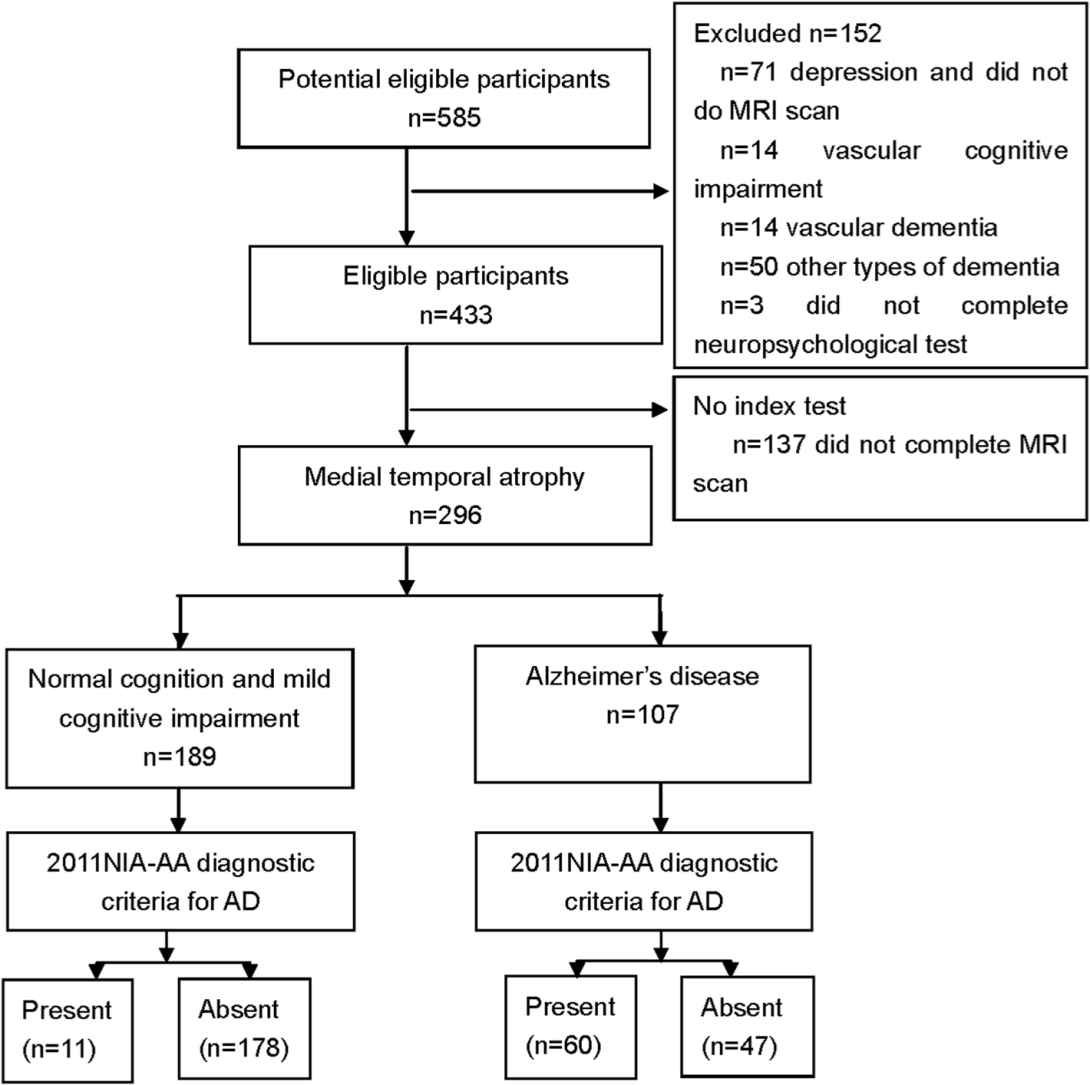
Standard study flow chart of medial temporal atrophy scale

A total of 585 subjects were enrolled in the study. Three patients were excluded because they did not complete the neuropsychological assessment, 137 patients did not complete MRI scan, 71 were diagnosed with depression and did not receive an MRI scan, 14 were considered as having vascular cognitive impairment (VCI), 14 exhibited vascular dementia (VaD), and 50 had a diagnosis of other types of dementia. Eighty seven were classified as normal cognition (NC), 138 as amnestic mild cognitive impairment (aMCI), and 71 as dementia of Alzheimer’s type (DAT). The standard study flow chart of MTA is shown in Fig. 1.

The characteristics of the NC, aMCI and DAT can be seen in Table 1. There were significant differences in age between the groups. The mean age of patients in the DAT group was significantly higher than that in the aMCI group and NC group (*P* < 0.01). All of the neuropsychological test scores in the DAT group were significantly lower than that in both the NC and aMCI groups (All *P* < 0.01).

**Table 1:** Comparison of demographic factors and neuropsychological assessment between three groups

	NC (CDR = 0.0)	aMCI (CDR = 0.5)	DAT (CDR ≥ 1.0)	x^2^/F	*p*
	*n* = 87	*n* = 138	*n* = 71		
Sex(F/ M)	57/30	81/57	28/43	14.14	< 0.01
Age	65.49 (7.54)	66.22 (8.65)	71.10 (9.66)^##&&^	17.69	< 0.01
Education	12.02 (3.15)	10.86 (3.52)*	11.43 (4.04)	5.80	0.055
MMSE	28.21 (2.94)	26.59 (1.93)**	15.28 (6.59)^##&&^	169.31	< 0.01
ISR	26.80 (9.91)	12.86 (6.91)**	5.13 (6.81)^##&&^	147.08	< 0.01
DSR	24.12 (10.76)	8.28 (7.22)	1.48 (3.76)^##&&^	156.04	< 0.01
CDT	3.82 (0.63)	3.60 (0.64)*	1.85 (1.46)^##&&^	115.14	< 0.01
TMT-A	55.07 (27.38)	71.703 (1.^25^)**	126.16 (37.50)^##&&^	80.45	< 0.01
TMT-B	101.07 (61.49)	137.04 (74.76)**	259.47 (82.62)^##&&^	69.31	< 0.01
ADL	14.25 (1.65)	14.49 (2.82)	27.50 (9.69)^##&&^	194.96	< 0.01
MTA	0.74 (0.62)	1.13 (0.92)*	2.26 (0.77)^##&&^	115.19	< 0.01
MTAi	4.33 (2.87)	3.42 (2.14)	3.39 (1.87)	3.91	0.141
GCA	0.46 (0.50)	0.59 (0.54)	0.90 (0.53)^##&&^	23.69	< 0.01
PA	0.61 (037)	0.65 (0.48)	0.59 (0.47)	1.12	0.571

Notes: *ADL* Instrumental Activities of Daily Living scale, *aMCI* amnestic Mild cognition impairment, *CDT* Clock drawing test, *DAD* Dementia of Alzheimer’s type, *DSR* Delayed story recall of the adult memory and Information processing battery, *GCA* global cortical atrophy scale, *ISR* Instant story recall of the adult memory and Information processing battery (AMIPB), *MTA* medial temporal atrophy scale, *MTAi* medial temporal atrophy index, *MMSE* Mini-mental state examination, *NC* Normal cognition, *TMT-A* Trail making test part A, *TMT-B* Trail making test part B, *PA* posterior atrophy.**P < 0.01 NC VS aMCI, *P < 0.05 NC VS aMCI; ^##^ *P* < 0.01 NC VS AD, ^&&^ *P* < 0.01 aMCI VS AD

### Influence of demographic and neuropsychological test on the visual rating scales

We entered age, years of education and neuropsychological scale into a multiple linear regression analysis with the visual rating scale score as the dependent variable respectively. We correlated visual rating scales with the MMSE, DSR,TMT,CDT, ADL and age across all subjects in the study. There were significant correlations between age and MTA (r = 0.029,*P* = 0.002), and MMSE had significant impacts on the mean MTA score(r = − 0.071, *P* = 0.006), whilst age was also associated with the GCA and PA score. The mean MTAi did not correlate with demographic and neuropsychological assessment scale.

### Cutoff scores and diagnostic value of visual rating scale for discriminating DAT from NC

**Fig. 2:**
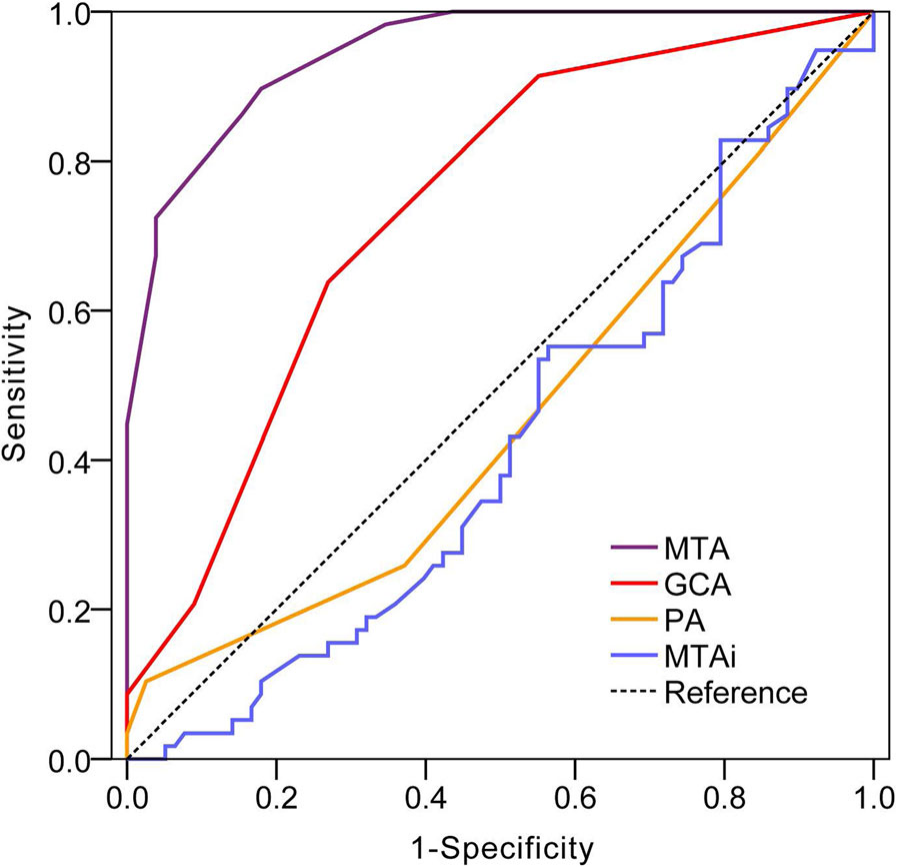
Receiver operating characteristic curve of visual rating scales in the differentiation of dementia of Alzheimer’s type from normal cognition. Notes: MTA = medial temporal atrophy; PA = posterior atrophy rating scale; GCA = global cortical atrophy scale; MTAi = medial temporal-lobe atrophy index

Using a ROC curve, we compared NC subjects with those diagnosed with DAT (Fig. 2). The area under the curve (AUC) was 0.42(95% CI:0.32–0.52) for MTAi, 0.46(95% CI:0.36–0.56) for PA, 0.74 (95% CI:0.65–0.82) for GCA and 0.92(95% CI:0.89~0.96) for MTA respectively. The MTAi and PA score showed relative low accuracy in distinguishing DAT from NC, and these two scales were not suitable for screening DAT. The GCA showed a sensitivity of 63.8% and specificity of 73.1% with the cutoff score of 1. Optimal GCA cut-off values for the age ranges 50–64, 65–74, 75–84 were: ≥0.5, ≥1.0, ≥ 1.0, the sensitivity and specificity were 84.6 and 47.4%; 52.4 and 77.8%, 79.2 and 53.8%.

The MTA scale showed the best value for diagnosis among the four rating scales. An MTA cutoff score of 1.5 yielded an optimal sensitivity (84.5%) and specificity (83.3%) for discriminating DAT and NC. The discrimination between NC and DAT stratified by age was also calculated, the AUC was 0.88 (0.78~0.98) for the 50–64 year group, 0.95 (0.89~1.00) for the 65–74 year group, 0.75 (0.63~0.93) for the 75–84 year group, optimal MTA cut-off values for the age ranges 50–64, 65–74, 75–84 were: ≥1.0, ≥1.5, ≥ 2.0, the sensitivity and specificity were 92.3 and 68.4%; 90.4 and 85.2% 70.8 and 82.3%.

We compared the diagnostic value between the Scheltens’ cutoff criteria with our study’s new cutoff criteria for discriminating DAT from NC (Table 2). Scheltens’ cutoff was reported as ≥2 points for ≤75 years, and ≥ 3 points for > 75 years. And the sensitivity and specificity was 60 and 95.6% respectively in the total subjects group, in the age ranges ≤75 years and > 75 years, the sensitivity and specificity were 70.7 and 95.5%, 40.7 and 74.6% respectively.

**Table 2:** Diagnostic value of visual rating scale for discriminating dementia of Alzheimer’s type from normal cognition

Scales	Criteria	Age group	Cutoffs	AUC(95%CI)	Sen(%)	Spe(%)	FNR(%)	Youden index	PPV(%)	NPV(%)
MTAi		–	–	0.42 (0.32–0.52)	–	–	**–**	–	–	–
PA		–	–	0.46 (0.36–0.56)	–	–	**–**	–	–	–
GCA		Total	1	0.74 (0.65–0.82)	63.8	73.1	36.2	0.369	49.0	80.9
		50–64	1	0.69 (0.54–0.83)	53.8	77.9	46.2	0.317	48.4	80.0
		65–74	0.5	0.74 (0.59–0.88)	90.5	48.1	9.5	0.386	59.1	68.9
		75–84	1	0.69 (0.50–0.88)	79.2	53.8	20.8	0.33	59.7	69.2
MTA	Tian et al’ s criteria	Total	1.5	0.92 (0.89~0.96)	84.5	79.1	15.5	0.636	56.1	94.2
		50–64	1.0	0.88 (0.78~0.98)	92.3	68.4	7.7	0.607	36.1	96.7
		65–74	1.5	0.95 (0.89~1.00)	90.4	85.2	9.6	0.756	49.8	96.7
		75–84	2.0	0.75 (0.63~0.93)	70.8	82.3	29.2	0.538	42.1	90.3
	Scheltens’ criteria	Total			60.0	95.6	40.0	0.556	74.8	88.7
		≤ 75	2.0		70.7	95.5	29.3	0.662	63.4	91.5
		> 75	3.0		40.7	74.6	59.3	0.153	25.1	80.5

Notes: *Sen* Sensitivity, *Spe* Specificity, *FNR* False negative rate, *PPV* Positive predictive values, *NPV* Negative predictive value, *AUC* Area under curve, *CI* Confidence intervals

### Visual rating scale for discriminating aMCI from NC

**Fig. 3:**
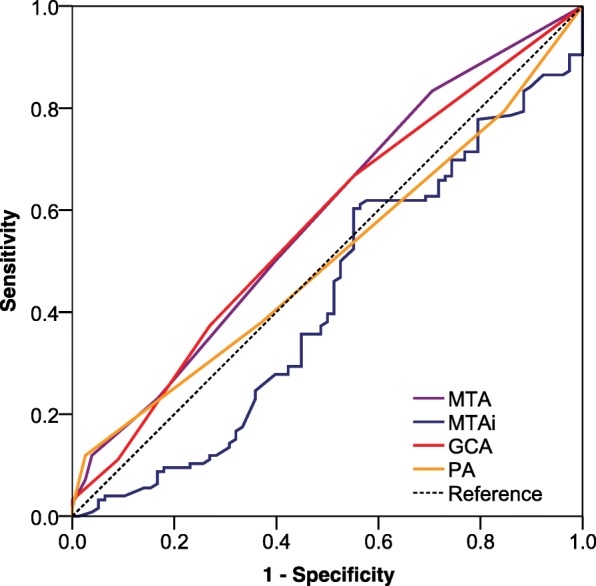
Receiver operating characteristic curve of visual rating scales in the differentiation of aMCI from normal cognition. Notes: MTA = medial temporal atrophy; PA = posterior atrophy rating scale; GCA = global cortical atrophy scale; MTAi = medial temporal-lobe atrophy index; aMCI = amnestic mild cognitive impairment

ROC analysis was performed to provide diagnostic values of visual rating scale for distinguishing subjects with aMCI from NC (Fig. 3). The AUC was 0.598 (95% CI: 0.522–0.673) for MTA, and 0.585(95%CI:0.504–0.665) for PA, 0.570 (95%CI: 0.490–0.651) for GCA, 0.424(95% CI:0.342–0.506) for MTAi. All of the scales showed relatively lower diagnostic values for aMCI.

### Diagnostic value of visual rating scale for discriminating DAT from aMCI

**Fig. 4:**
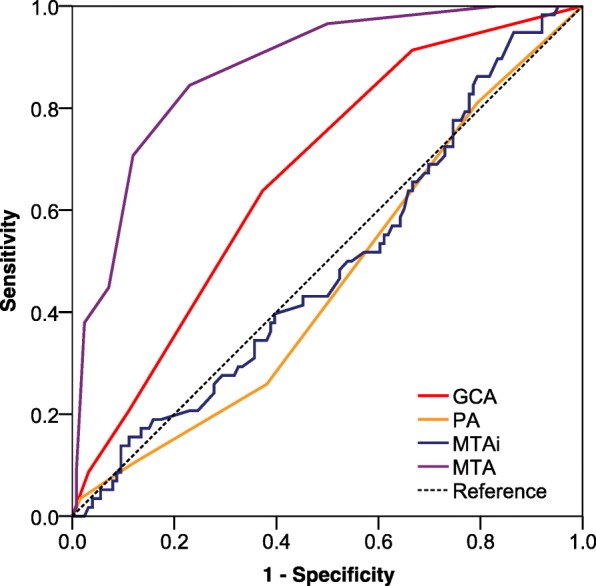
Receiver operating characteristic curve of visual rating scales in the differentiation of dementia of Alzheimer’s type from aMCI. Notes: MTA = medial temporal atrophy; PA = posterior atrophy rating scale; GCA = global cortical atrophy scale; MTAi = medial temporal-lobe atrophy index; aMCI = amnestic mild cognitive impairment

When the visual rating scales were used to detect DAT from aMCI using a ROC curve, the AUC was 0.49 (95% CI:0.404–0.582) for MTAi, 0.46(95% CI:0.374–0.550) for PA, 0.67(95%CI:0.590–0.751) for GCA and 0.87 (95% CI:0.824–0.928) for MTA respectively. When the cutoff score was 1.5 for MTA to discriminating aMCI from DAT, a sensitivity of 84.5% and specificity of 77.0% was obtained (Fig. 4).

## Discussion

In this study, we have compared the diagnostic value of visual rating scales of MTA, PA, GCA and MTAi for distinguishing DAT from NC, and correlation with age, education and neuropsychological assessment scales. The results showed that MTA was a useful tool for assessing medial temporal atrophy in DAT, age contributed to MTA score.

Studies have showed that MTA, PA and GCA correlated with age [11, 12]. Older subjects showed more serious atrophy of medial temporal, posterior and global cortical regions, which were also found in other studies [13]. This was consistent with the findings of Dekaban AS [22], who showed that brain weight began to decline at the age of 45 to 50 and reached its lowest values after age 86. Therefore, age should be taken into consideration when defining atrophy of the hippocampus.

In this study, the MTA was significantly correlated to MMSE scores, higher MTA score correlated with lower MMSE scores, this is consistent with the previous study of [7], who showed that neuropathological changes underlying AD first occurred in the medial temporal lobe [23, 24], therefore, MTA may be more sensitive than other visual rating scales for early stage AD. Other study have shown that MTA was associated with memory impairment in prodromal AD [25], whereas volumes of the left temporo-parietal regions were correlated with performance in naming and praxia. Left frontal lobe atrophy was associated with verbal fluency [26]. One study based on postmortem hippocampal volume on MRI was more consistent with Alzheimer neuropathology than the clinical diagnosis or measures of cognition, implying that hippocampal volume on MRI is a better predicator [27].

In contrast to MTA, the MTAi showed no relationship with demographic factors, and also there was no difference between the DAT, aMCI and NC groups. This was inconsistent with recent studies on MTAi [15, 27], which showed that MTAi had differential values between short series of patients and healthy control, aMCI and DAT groupings. The possible reason may be that MTAi was calculated on a single coronal slice, and the selection procedure may have impacted the results.

Previous studies have reported that when the cutoff was designated at ≥2.0 for ≤75 yrs., and ≥ 3.0 for > 75 yrs. of MTA for discrimination of AD from NC, a sensitivity of 81% and a specificity of 67% was obtained [7], this method correctly identified 60% of AD patients (sensitivity) and 95.6% of controls (specificity) in our Chinese population, the FNR was 40%. In our study, sensitivities of 84.5% and specificities of 79.1% were obtained for discriminating DAT from NC, and the FNR was 15.5%, thereby improving the performance on diagnosis of DAT by 24.5% compared with the previous Scheltens’ cutoff criteria.

Another study conducted in a Netherlands population, employed a new MTA cut-off value for the age ranges < 65, 65–74, 75–84 and ≥ 85 were: ≥1.0, ≥1.5, ≥ 2.0 and ≥ 2.0, corresponding values of sensitivity and specificity were 83.3 and 86.4%; 73.7 and 84.6%; 73.7 and 76.2%; and 84.0 and 62.5%^13^. This was also consistent with our results.

In this study, the MTA showed poor accuracy for discriminating aMCI from NC, and this result indicated that the new age-based cutoff score may be more suitable for the diagnosis and differential diagnosis typical AD phenotype in clinical practice.

PA showed lower diagnostic value for discriminating DAT from NC, and this was consistent with Ferreira D., et al. [11], the AUC was 0.567 when PA was used to discriminate AD from NC [12], the reason may be that most of the AD subjects enrolled in this study had a typical AD phenotype, which always showed atrophy of the medial temporal lobe, rather than the posterior atrophy.

## Limitation

The limitations of our study is shown as follows: firstly, lack of pathological confirmation of the diagnoses; secondly, the sample size of patients was relatively small, especially the DAT group; moreover, all subjects were enrolled from a memory clinic, the subjects identified as normal cognition may not be representative of the normal healthy population; thirdly, the most AD subject enrolled in this study was amnestic AD, and we did not enroll atypical AD subjects, which could have influenced the PA results; lastly, we only calculated the cutoff scores of subjects among 50–84 years old patients, the cutoff scores of subjects older than 85 years age group was not calculated. Hence, further studies need to be conducted on a larger scale, using population based healthy controls to evaluate the predictive value of MTA.

## Conclusion

The new age-based cutoffs of MTA scale showed a significantly better diagnostic accuracy for detecting AD than the current gold-standard with relatively high sensitivity and specificity within Chinese population.

## Abbreviations

AD: Alzheimer’s disease
aMCI: Amnestic mild cognitive impairment
CDR: Clinical Dementia Rating scale
CDT: Clock drawing test
DAT: Dementia of Alzheimer’s type
DSR: Adult Memory and Information Processing Battery story recall
FNR: False negative rate
GCA: Global cortical atrophy scale
HAMD: Hamilton Depression Scale
HIS: Hachinski Ischemia scale
IADL: Instrumental Activities of Daily Living scale
MMSE: Mini-mental State Examination
MRI: Magnetic resonance imaging
MTA: Medial temporal atrophy
MTAi: Medial temporal-lobe atrophy index
NC: Normal cognition
PA: Posterior atrophy rating scale
ROC: Receiver operating characteristic
TMT: Trail Making Test
VaD: Vascular dementia
VCI: Vascular cognitive impairment
VRS: Visual rating scales
